# Multiple forensic insights for human identification using single teeth with applicability to advanced postmortem remains

**DOI:** 10.1038/s41598-026-62088-z

**Published:** 2026-08-19

**Authors:** Sayaka Yamada, Hisako Saitoh, Kanju Saka, Saki Minegishi, Toru Moriya, Mirei Takeyama, Hajime Utsuno, Shuuji Namiki, Muhammad Garry Syahrizal Hanafi, Nanami Aoki, Yohsuke Makino, Hirotaro Iwase, Fuyuki Tokanai, Koichi Sakurada

**Affiliations:** 1 Department of Forensic Dentistry, Graduate School of Medical and Dental Sciences, Institute of Science Tokyo, 1-5-45 Yushima, Bunkyo-ku, Tokyo, 113- 8510 Japan; 2 Department of Forensic Medicine, Graduate School of Medicine, The University of Tokyo, 7-3-1 Hongo, Bunkyo-ku, Tokyo, 113-0033 Japan; 3 Center for Accelerator Mass Spectrometry, Yamagata University, 19-5 Kanakame, Kaminoyama, 999-3101 Yamagata Japan; 4 Department of Legal Medicine, Graduate School of Medicine, Chiba University, 1-8-1 Inohana, Chuo-ku, Chiba, 260-8670 Japan

**Keywords:** DNA typing, Racemization, Radiocarbon dating, Stable isotopes, Toxicological analysis, Biological techniques, Genetics, Medical research

## Abstract

Prolonged postmortem intervals cause soft tissue loss, complicating identification in disasters and criminal investigations. We used a single mandibular first premolar from bodies (*n* = 15) with a long postmortem interval for simultaneous ^14^C and δ^13^C isotope analysis, amino acid racemization analysis, DNA profiling, and toxicological analysis. ^14^C analysis gave multiple estimated birth years, including actual birth year, in 14 of 15 cases, and amino acid racemization analysis estimated ages at death within a margin of mean error of − 5.82 ± 6.87 years (mean absolute error (MAE) = 7.93, *r* = 0.86). Integrating both results increased the year of birth estimation accuracy (0.57 ± 6.36 years, MAE = 5.01, *r* = 0.95). Estimated region based on δ^13^C analysis did not contradict known individual information. DNA analysis estimated sex in all cases, and a sufficient number of autosomal short tandem repeat (STR) loci for individual identification was detected in 12 cases. Mitochondrial DNA typing and Y-STR analysis were also successful. Toxicological analysis of dental pulp detected drugs consistent with autopsy screening results and drug use information. The findings suggest that various identifying information can be obtained from a single tooth, representing a novel, useful strategy for rapidly narrowing potential individuals.

**Supplementary Information:**

The online version contains supplementary material available at 10.1038/s41598-026-62088-z.

## Introduction

In the aftermath of major disasters and during routine criminal investigations, personal identification is an essential task. When postmortem damage or decomposition is limited, identification can often be achieved through facial features, personal belongings, and witness information. Similarly, when a body is found inside a residence, such as in the aftermath of a fire, the owner can be presumed, and identification is usually achieved quickly through dental findings or DNA typing. However, in cases where a long time has elapsed since death, decomposition is usually advanced. In cases of skeletal remains found outdoors, supplementary information, such as clothing and personal belongings, is often lost, and biological samples required for blood or soft tissue analyses are usually no longer available. In such cases, investigators have traditionally relied on information obtained from bones and teeth to narrow down the identity of the deceased. Sex, age, and stature can be estimated from bone morphology and bone length^1^. Although these biological profile estimates are relatively reliable when the entire skeleton is preserved, age estimation based on skeletal morphology may still have a margin of error of 20–30 years^2^.

Teeth provide useful information not available from bones, such as an individual’s living conditions during their lifetime from detailed dental records obtained via visual examination and X-rays. However, estimating sex is difficult, and the accuracy of age estimation, particularly for middle-aged and older individuals, is comparable to that of bone^3,4^. In addition, bone has a porous structure with numerous spaces for blood vessels and bone marrow, making it more susceptible to the effects of external environmental factors, such as decomposition, soil-derived microorganisms, moisture, and chemical substances^5^. In contrast, the crown of the tooth is covered by enamel, the hardest tissue in the human body, and the root is protected further by cementum, which limits the penetration of exogenous substances into internal tissues, such as the dental pulp^6^. Consequently, teeth are more resistant to decomposition and external contamination and exhibit superior long-term preservation compared with bone, making them a valuable source for DNA analysis^7,8^. Metabolic changes in teeth after formation are minimal because teeth do not undergo continuous remodeling, in contrast to bone^9^. The timing of formation is clearly defined for each type of tooth, and thus it is possible to obtain information associated with specific time periods. These structural characteristics mean that various analytical methods using teeth, such as radiocarbon dating, stable isotope analysis, amino acid racemization (AAR) analysis of dentin, and toxicological analysis, have been reported, offering the potential to provide more accurate information than was previously possible.

In radiocarbon dating, the estimation of birth year based on ^14^C analysis relies on the fact that the ^14^C concentration in enamel reflects the atmospheric ^14^C concentration at the time of crown formation^10^. Therefore, it is possible to estimate the time of crown formation based on the characteristic bomb curve, which features a sharp rise in atmospheric ^14^C concentrations caused by nuclear tests during the Cold War, followed by a decline peaking in 1963. Once crown formation is complete, enamel ceases carbon incorporation from the external environment, and its composition remains virtually unchanged for the rest of a person’s life. Therefore, year of birth (YOB) can be estimated with high precision by subtracting the age at crown completion from the estimated time of crown formation^11^.

Stable isotope analysis is a method for estimating dietary environments during tooth formation using stable isotope ratios recorded in tooth enamel and dentin as indicators^12^. ^13^C incorporated from C3 plants, such as rice, and C4 plants, such as corn, is preserved in enamel and dentin and provides a useful source of information for estimating the dietary habits and growth environments during tooth formation.

AAR analysis of dentin estimates age based on changes in the isomeric ratio of amino acids in biological tissues. Amino acids in living organisms normally exist as the L-form, but as racemization progresses with aging, the L-form is replaced by the D-form. In particular, aspartic acid has a fast racemization rate, and Helfman et al.^13^ reported a high correlation between the racemization rate of aspartic acid in dentin and age. Ohtani et al.^14,15^ reported that AAR analysis using a calibration curve for the same tooth type could estimate age at death with an accuracy of ± 3 years.

When decomposition is advanced, it is often difficult to determine the cause of death accurately. With skeletal remains, it is extremely difficult to determine whether drugs or toxic substances were involved in the cause of death. Although toxicological analysis using bone is effective^16,17^, the forensic interpretation of the concentrations requires careful consideration owing to differences in the distribution of drugs within bone and the effects of postmortem changes. Consequently, toxicological analysis has been performed using teeth, which are less susceptible to external environmental influences. Bianchi et al.^18^ reported that using teeth may be feasible based on pilot toxicological analyses from a drug-related death.

Although teeth can provide valuable information, combining multiple analytical methods can lead to more comprehensive insights compared with a single approach. Alkass et al.^19,20^ reported using several types of intact teeth to improve the accuracy of identity-related information by integrating analytical results, such as individual identification via DNA analysis, life history estimation via stable isotope analysis, and birth year estimation via radiocarbon analysis. Hulst et al.^21^ reported multiple analyses on teeth and toenails from the same individual. They performed DNA analysis on the dental pulp, isotope analysis on the enamel, ^14^C analysis and toxicological analysis on the root, and tooth cementum annulation (TCA) analysis on the root cementum to estimate age. They also performed all analyses except TCA analysis on toenails. By evaluating the results from teeth and nails, the researchers extracted accurate personal identification information.

Previous studies have primarily used samples from bodies with short postmortem intervals and minimal postmortem changes, and no studies have reported the use of samples from bodies with medium- to long-term postmortem intervals. In addition, biochemical analyses have generally been performed using multiple teeth or a combination of teeth and other biological samples.

In this study, with the aim of applying our approach to actual forensic cases, we performed multiple analyses on a single tooth to evaluate the potential for obtaining the maximum amount of information from a minimal sample. The mandibular first premolar was selected as the target tooth for the following reasons. Tooth germ formation in the first premolar begins immediately after birth and the crown formation period ends approximately 5–6 years later; thus, the first premolar is more likely to reflect isotopes incorporated around the YOB compared with molars, which have a longer formation period. In premolars, the pulp chamber is located in the center of the tooth. Therefore, when longitudinal sections are prepared, the pulp is contained within the central section. For this reason, analyses involving the pulp can be performed efficiently by using this section. In addition, premolars are single-rooted and relatively easy to extract, and are in a position that is covered by the lips and maxillary teeth; thus, samples can be collected with a small effect on appearance. Consequently, a single tooth may provide valuable clues for identification beyond those obtainable by conventional methods, including estimation of YOB, geographic origin, age at death, DNA typing, and assessment of drug use history.

## Materials and methods

### Samples

One mandibular first premolar was extracted from each of the 15 bodies, comprising 5 skeletal remains and 10 highly decomposed bodies that underwent forensic autopsy, resulting in a total of 15 teeth (case nos. 1–15) for analysis. The teeth selected were those on either the left or right side that showed no signs of caries or dental treatment. Details of the samples are shown in Table 1. At the time of autopsy, all subjects were unidentified; however, the identities of 14 subjects were subsequently confirmed. In the remaining case, although a potential individual has been identified based on the investigative information, definitive identification has not yet been achieved. In this study, the YOB was used to represent the birth year of each individual. The YOB obtained after identification was defined as the actual YOB, whereas the age from the actual YOB to the last confirmed date of survival based on the investigative information was defined as the assumed age at death (AAD). This study was approved by the Ethics Review Committee of the School of Dentistry, Institute of Science Tokyo (Approval No. D2023-028). All experiments were performed in accordance with relevant named guidelines and regulations.

**Table 1:** Sex, tooth number, actual year of birth (YOB), date last known to be alive, autopsy date, assumed age at death (AAD), and postmortem condition of the body for each case.

Case no.	Sex	Tooth no.	Actual YOB	Date last known to be alive	Autopsy date (year)	AAD	Condition of the body
1	M	34	1957.74	2019.39	2019.43	61.65	Advanced decomposition
2	M	34	1959.45	2020	2020.63	61	Skeletal remains
3	M	44	1952.67	2015.76	2015.79	63.09	Advanced decomposition
4	M	44	1960.80	2017.15	2017.35	56.35	Advanced decomposition
5	M	34	1956.57	2015.25	2015.84	58.68	Skeletal remains
6	F	34	1954.20	2012.98	2014.08	58.78	Skeletal remains
7	M	44	1966.95	2017.55	2017.60	50.6	Advanced decomposition
8	F	44	1952.32	2025.44	2025.50	73.12	Advanced decomposition
9	F	34	1990.70	2025.39	2025.56	34.69	Advanced decomposition
10	F	34	1971.04	2025.42	2025.53	54.38	Advanced decomposition
11	F	44	1986.51	2013.58	2013.64	27.07	Advanced decomposition
12	M	44	1948.59	2011	2014.65	63	Skeletal remains
13	M	44	1967.12	2016.28	2016.80	49.16	Advanced decomposition
14	M	44	1944.00	2004.42	2017.85	60.42	Skeletal remains
15	M	44	Unknown (Estimated 1972)	2013.12	2013.30	41 (Estimated age)	Advanced decomposition

For cases no. 2 and no. 12, the date last known to be alive is presented as year only because the exact date was unknown. AAD was calculated as the interval between the actual YOB and the date last known to be alive. For case no. 15, based on investigative information, the individual was estimated to be a 41-year-old male at the time, suggesting a birth year around 1972; however, the exact date of birth is unknown.

## Sample preparation

**Fig. 1:**
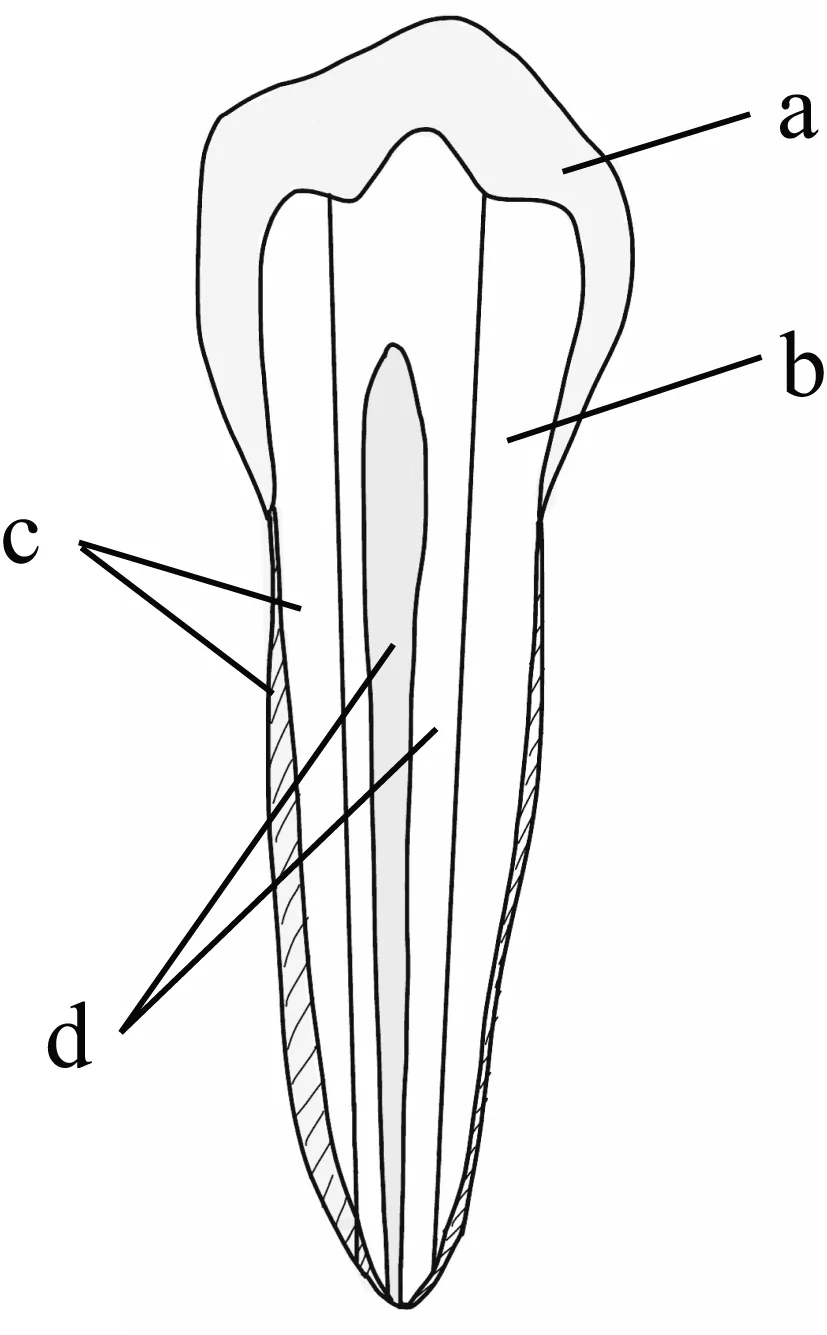
Schematic examples of sample collection sites (cross-sectional view of a tooth). a: Enamel used for ^14^C and δ^13^C analysis. b: Dentin used for amino acid racemization analysis. c: Dentin and cementum used for DNA analysis. d: Pulp and dentin used for toxicological analysis.

^***14***^***C and δ***^***13***^***C analysis***.

The teeth were washed with 99.5% ethanol and distilled water, and debris on the tooth surfaces was removed using a dental bur. The teeth were left to dry at room temperature overnight. After drying, the teeth were sectioned using an automatic precision cutting machine (IsoMet; Buehler, Lake Bluff, IL, USA) to produce longitudinal sections about 1 mm thick parallel to the tooth axis (Supplementary Fig. S1). Figure 1 shows the approximate sampling locations used for each analysis. At least one section was allocated for each analysis. When the tooth was large enough to yield a sufficient number of sections, two sections were used from each site. From each section, only the enamel was roughly excised using the cutting machine (a in Fig. 1), and this was used for ^14^C and δ^13^C analyses. Based on reports that cementum preserves DNA well^22^, for the mesial or distal regions (b and c in Fig. 1), one section from the side with the largest area of cementum was used for DNA analysis. One or two sections from the opposite side were used for AAR analysis of dentin. One or two sections from the central region containing the pulp chamber were used for toxicological analysis (d in Fig. 1).

For the collected enamel samples (a in Fig. 1), residual dentin was removed using a dental bur under a stereomicroscope, and only the enamel was used as the sample. The enamel was sealed in a tin cup, combusted using an elemental analyzer (Variomicro Cube; Elementar, Langenselbold, Germany), and converted into CO_2_ gas. 10% of the total gas was introduced into a mass spectrometer (IRMS; Elementar) to measure the δ^13^C value. The remaining 90% was introduced into a glass vacuum line and graphitized via a hydrogen reduction reaction using an iron catalyst^23^. The ^14^C concentration of approximately 1 mg of the resulting graphite was measured using an accelerator mass spectrometer (1.5SDH-1; National Electrostatics Corp., Middleton, WI, USA)^24^. The standard sample was HOxII (SRM-4990 C; National Institute of Standards and Technology, Gaithersburg, MD, USA), and IAEA-C1 was used for background evaluation. The ^14^C/^12^C ratio of the measurement data was calculated by subtracting the background value of IAEA-C1. The ^13^C/^12^C ratio was measured simultaneously and used for the *δ*^13^C correction of isotopic fractionation. The estimated YOB was calculated based on the results. According to Daito et al.^25^, the age at crown completion for the mandibular first premolar in Japanese individuals for the left and right mandibular sides were 5.29 and 5.33 years in males, and 4.98 and 4.99 years in females, respectively. In this study, these values were used as tooth formation time (TFT). The estimated YOB was defined in three steps. First, the percent modern carbon (pMC) value was calibrated to obtain the calibrated formation range, which was defined as the estimated tooth YOB in this study. Second, the estimated YOB ranges were calculated by subtracting TFT from the estimated tooth YOB. Third, following a practical approach similar to that of Kunita et al.^26^, the representative value was calculated as (upper limit + lower limit)/2 and the intervals as (upper limit − lower limit)/2, and the estimated YOB was expressed as the representative value ± intervals. Subsequent analyses were based on these representative values and intervals. These representative values are not probability-weighted means of the calibrated probability distributions.

In ^14^C analysis, because of the characteristics of the atmospheric ^14^C concentration bomb curve, which peaks in 1963, the same ^14^C value may correspond to the rising phase or the declining phase, resulting in multiple candidate estimated birth years^20,27^. In this study, the estimated year of death was calculated by adding the estimated age at death, obtained through AAR analysis, to the estimated YOB. The date that was consistent with the final confirmed date of survival obtained from investigative information, as well as the autopsy date, was taken as the final estimated YOB.

## Measurement of AAR

Using one or two dentin sections from the mesial or distal side (b in Fig. 1), sample pretreatment and amino acid extraction were performed with reference to the method described by Minegishi et al.^28–30^. For sample pretreatment, the dentin sections were ultrasonically cleaned in 0.2 M HCl (5 mL) and distilled water. The samples were defatted using ethanol (5 mL) and diethyl ether (5 mL), and left to dry overnight. The dried sections were pulverized using a pulverizer (Multi-beads Shocker; Yasui Kikai, Osaka, Japan), and the powder (approximately 10 mg) was transferred to a test tube. Hydrolysis was performed by adding 6 M HCl (5 mL) and heating at 100 °C for 6 h. After hydrolysis, HCl was completely evaporated using an evaporator (CCA-1112 A, SB-1200, and N-1300; Tokyo Rikakikai, Tokyo, Japan). Distilled water (5 mL) was added to the residue to dissolve it, and the solution was dropped onto a resin column packed with cation exchange resin. The amino acids were eluted with 2 N ammonia (10 mL), and the eluate was completely evaporated using an evaporator. A solution of acetyl chloride and isopropanol (2 mL, 1:4 v/v) was added to the test tube, heated at 100 °C for 30 min, and completely dried with nitrogen gas. Methylene chloride (800 µL) and anhydrous fluoroacetic acid (200 µL) were added, and after trifluoroacetic acid treatment at room temperature for 30 min, the mixture was completely dried with nitrogen gas. The mixture was reconstituted with ethyl acetate (200 µL), and 1 µL of this solution was analyzed using a gas chromatograph (7890B; Agilent Technologies, Santa Clara, CA, USA) equipped with an FID detector. The column used for analysis was CP-Chirasil L-VAL (Agilent Technologies; length 25 m, diameter 0.25 mm, film 0.12 μm), and the D-Asp/L-Asp ratio was measured as the racemization rate based on the peak integration values of D-Asp and L-Asp.

Separate from the samples in this study, six mandibular first premolars (five from males, one from a female; three from the left side, three from the right side) aged 25–72 years were used to create a calibration curve based on the relationship between the racemization ratio and age. The estimated age at death was calculated by substituting the racemization ratios of each sample in this study into the calibration curve. All reagents used were commercially available, highest-grade products (Fujifilm Wako Pure Chemical Corporation, Osaka, Japan).

## DNA typing

### DNA extraction

DNA extraction was performed using the TBONE EX Kit (DNA Chip Research Inc., Kawasaki, Japan) on one or two sections (c in Fig. 1) containing the highest proportion of cementum. Although the TBONE EX Kit protocol specifies the use of the QIAamp Mini Spin Column from the QIAamp DNA Mini Kit (Qiagen, Hilden, Germany)^31^, we used the QIAamp MiniElute spin column included with the QIAamp DNA Investigator Kit (Qiagen). Low TE Buffer (10 mM Tris-HCl, 0.1 mM EDTA-2Na [pH 8.0]) was used for the DNA solution (final volume of 40 µL).

To assess the presence of DNA contamination, we pre-screened the DNA profiles of the researchers who performed sample pretreatment, DNA extraction, and DNA analysis, as well as those of personnel entering and exiting the DNA analysis workspace.

### DNA quantification by real-time PCR

DNA quantification was performed using a PCR system (QuantStudio 5; Thermo Fisher Scientific, Waltham, MA, USA) with Quantifiler HP and Trio DNA Quantification Kits (Thermo Fisher Scientific) for human DNA quantification. The extracted DNA solution (2 µL) was used for PCR according to the kit manufacturer’s manual^32^. HID Real-Time PCR Analysis Software ver. 1.3 (Thermo Fisher Scientific) was used for data analysis. In addition to DNA quantification, the degradation index (DI) and the internal PCR control (IPC) Ct value were measured. The DI value is automatically calculated from the concentration ratio of the small DNA target to the large DNA target on autosomes. If this ratio is ≤ 1, the DNA is undegraded; if the ratio is > 1, the sample is substantially degraded. The IPC Ct value was used to evaluate PCR inhibition. An IPC Ct value greater than the normal range (Ct: 25.5–30.5) indicated the presence of PCR-inhibiting substances. The degree of DNA degradation and PCR inhibition in each sample was assessed to evaluate the quality of the extracted DNA.

## STR typing and Y-STR typing

For short tandem repeat (STR) typing, we used the GlobalFiler PCR Amplification Kit (Thermo Fisher Scientific), which enables simultaneous analysis of 21 autosomal STR loci, one Y-STR locus (DYS391), and Y-indel and amelogenin types. A PCR system (ProFlex; Thermo Fisher Scientific) was used for PCR, with 30 cycles; all other conditions followed the manufacturer’s instructions^33^. After capillary electrophoresis of the PCR amplification products on a genetic analyzer (SeqStudio; Thermo Fisher Scientific), Gene Mapper ID-X software (Thermo Fisher Scientific) was used to determine STR genotypes for loci where peaks of 50 RFU or more were obtained. Regarding the feasibility of individual identification via STR typing, based on reports on the Japanese population in the 2020 census, we determined that individual identification is possible when nine or more autosomal STR loci are detected^34^. Based on the results of sex determination using the DYS391, Yindel, and Amelogenin genes with the GlobalFiler PCR Amplification Kit, analysis was performed on extracted DNA (10 µL) using the YFiler Plus PCR Amplification Kit (Thermo Fisher Scientific) for 10 samples (case nos. 1–5, 7, and 12–15) that were likely to be of male origin. The PCR cycle number was set to 30, and all other conditions followed the manufacturer’s manual^35^. Analysis was performed under the same conditions as for STR typing, and the detection rate was defined as the percentage of loci detected out of the total 27 loci.

## Mitochondrial DNA typing

For mitochondrial DNA (mtDNA) typing, the HV1 region (16,086–16,362 bp according to Anderson et al.^36^ and the HV2 region (68–315 bp according to Anderson et al.^36^, which are hypervariable regions of mtDNA highly specific to individuals, were used for analysis.

Sequencing analysis of the HV1 and HV2 regions was performed using the extracted DNA (2.0 µL) as a template. The PCR reaction mixture consisted of 10× PCR buffer (5.0 µL, Toyobo Co., Ltd., Osaka, Japan), 2 mM dNTPs (5.0 µL), forward primer (2.0 µL, 10 pmol/µL), reverse primer (2.0 µL, 10 pmol/µL), and thermostable DNA polymerase (1.25 Units, Blend Taq-Plus, Toyobo Co., Ltd.). Nuclease-free water was added to a final volume of 50 µL.

In the HV1 region, to amplify the first half of the region from 15,977 to 16,255 bp, FN15977 (5′–CCACCATTAGCACCCAAAGC–3′)^37^ and R16255 (5′–CTTTGGAGTTGCAGTTGATG–3′) were used as the reverse primer. To amplify the latter region (16,140–16,410 bp), F16140 (5′–TACTTGACCACCTGTAGTAC–3′) was used as the forward primer and R16410 (5′–GAGGATGGTGGTCAAGGGAC–3′)^38^ as the reverse primer.

In the HV2 region, to amplify the first half (36 to 339 bp), we used F36 (5′–GAGCTCTCCATGCATTTGGT–3′) as the forward primer and R339 (5′–GTGTTTAAGTGCTGTGGCCAG–3′) as the reverse primer, and for the amplification of the latter region (257 to 429 bp), F257 (5′–ACAGCCACTTTCCACACAGA–3′) was used as the forward primer and L429 (5′–CTGTTAAAAGTGCATACCGCC–3′)^39^ as the reverse primer. Primer sequences not cited in the references were designed in this study.

The PCR reaction was performed using the ProFlex PCR System (Thermo Fisher Scientific). After an initial denaturation step at 94 °C for 2 min, the reaction cycle consisted of 40 cycles of 94 °C for 30 s, 60 °C for 30 s, and 72 °C for 1 min, followed by a final extension at 72 °C for 7 min, after which the mixture was cooled to 10 °C. PCR amplification was verified by electrophoresis on a 2% agarose gel.

Each PCR amplification product was purified using the QuickStep 2 PCR Purification Kit (Edge BioSystems, Inc., Gaithersburg, CA, USA), and cycle sequencing was performed on these purified products using the BigDye Terminator v1.1 Cycle Sequencing Kit (Thermo Fisher Scientific) according to the protocol.

The cycle sequencing reaction products were purified using the BigDye XTerminator Purification Kit (Thermo Fisher Scientific), followed by electrophoresis using a genetic analyzer (SeqStudio, Thermo Fisher Scientific). These sequencing data were analyzed using Sequencing Analysis Software ver. 8.0 (Thermo Fisher Scientific) to determine the nucleotide sequences.

For mtDNA genotyping, the nucleotide sequences of the HV1 and HV2 regions reported by Anderson et al.^36^ were used as reference sequences. The nucleotide sequence results were compared using SeqScape Software ver. 4.0 (Thermo Fisher Scientific), and the mtDNA genotype was determined based on the number and locations of nucleotide substitutions.

### Toxicological analysis

Using a central dentin section containing dental pulp (d in Fig. 1), the analysis was performed with minor modifications based on the methods of Hulst et al.^21^ and Klima et al.^40,41^. The sample was pulverized using a pulverizer (Multi-beads Shocker; Yasui Kikai). The powder (100 mg) was placed in a tube, and internal standard solution (10 µL) and extraction solution (600 µL) were added. The internal standard solution contained diazepam-D5 (4 µg/mL) and phenobarbital-D5 (200 µg/mL), and the extraction solvent was acetonitrile/ethanol/formic acid (90:10:0.075 v/v/v). The mixture was placed in an ultrasonic bath for 3 h, followed by overnight agitation on a rotary shaker to extract drugs and toxic substances. The next day, the sample was centrifuged at 9100*g* for 10 min, the supernatant was transferred to a separate tube, and the solvent was removed under a stream of nitrogen. The residue was redissolved in methanol (100 µL) and transferred to a vial. Each sample (5 µL) was analyzed by liquid chromatography-tandem mass spectrometry (LC-MS/MS; LCMS-8060NX, Shimadzu, Kyoto, Japan). Measurements were performed based on the Rapid Drug and Toxic Substance Screening System Version 3.01 (Shimadzu). Chromatographic separation was performed using a Shim-pack Velox SP-C18 column (100 × 2.1 mm, 2.7 μm; Shimadzu) equipped with a Shim-pack Velox EXP SP-C18 guard cartridge (Shimadzu), maintained at 40 °C. Mobile phase A consisted of 10 mM ammonium formate and 0.1% formic acid in water, and mobile phase B consisted of 10 mM ammonium formate and 0.1% formic acid in methanol. The flow rate was 0.3 mL/min. The gradient elution program was as follows: 5% B at 0 min; increased linearly to 95% B at 7.5 min; held at 95% B until 10.0 min; returned to 5% B at 10.01 min; and maintained at 5% B until 15.0 min. Mass spectrometry was performed in electrospray ionization mode under the following conditions: nebulizer gas flow rate, 3.0 L/min; drying gas flow rate, 10 L/min; heating gas flow rate, 10 L/min; interface temperature, 270 °C; desolvation line temperature, 250 °C; and heat block temperature, 400 °C. High-purity argon and nitrogen were used as collision and nebulizer gases, respectively. Data were acquired in the survey multiple reaction monitoring-dependent product ion scan mode. LabSolutions LCMS Ver. 5.128 SP2 software (Shimadzu Corporation) was used for data analysis.

This LC-MS/MS analytical method was developed for forensic toxicological analysis. Data analysis was performed by comprehensively evaluating the similarity between the product ion scan data of the samples and those of the reference standards in the library, the match in retention times, and the signal-to-noise ratio. This study examined whether the 251 drugs and toxic substances targeted by this analytical system were present in the samples (qualitative analysis).

## Results

^***14***^***C analysis***.

**Fig. 2:**
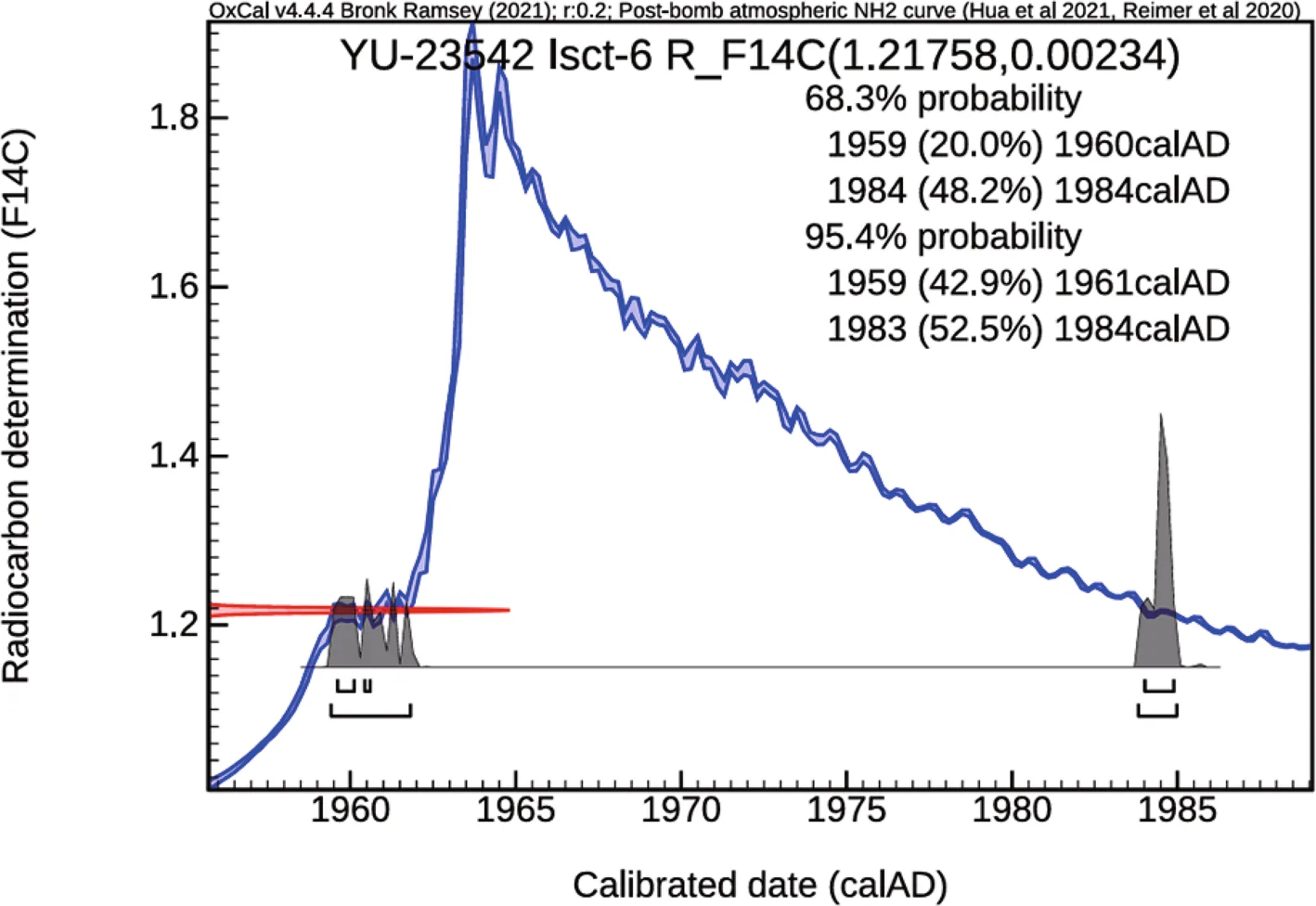
Example of ^14^C analysis of enamel. Bomb curve showing the ^14^C measurement results for enamel case no. 1.

The ^14^C analysis results are shown in Table 2. For case no. 1, the ^14^C value (pMC, ± 1σ) was 121.76 ± 0.23. The estimated tooth YOB calculated from these values was 1959.42–1961.82 and 1983.82–1984.98. Because this sample is the right mandibular first premolar of a male, subtracting the TFT of 5.29 years resulted in estimated YOBs of 1955.33 ± 1.20 and 1979.11 ± 0.58. Figure 2 shows the bomb curve for the radiocarbon dating of case no. 1.

**Table 2:** Results of ^14^C and δ^13^C analyses and estimated year of birth (YOB).

Case no.	δ^13^C (‰)	^14^C concentration (pMC ± 1σ)	Calibrated formation range (2σ) (Estimated tooth YOB)	TFT	Estimated YOB (range)	Estimated YOB
1	−15.9	121.76 ± 0.23	1959.42–1961.82 1983.82–1984.98	5.29	1954.13–1956.53 1978.53–1979.69	1955.33 ± 1.20 1979.11 ± 0.58
2	−16.6	156.89 ± 0.26	1968.16–1969.56	5.29	1962.87–1964.27	1963.57 ± 0.70
3	−19.6	101.21 ± 0.20	1955.40–1956.10	5.33	1950.07–1950.77	1950.42 ± 0.35
4	−17.5	151.58 ± 0.26	1969.92–1971.14 1971.36–1972.26	5.33	1964.59–1965.81 1966.03–1966.93	1965.20 ± 0.61 1966.48 ± 0.45
5	−20.1	119.60 ± 0.22	1959.10–1960.96 1985.70–1986.90 1987.34–1987.84	5.29	1953.81–1955.67 1980.41–1981.61 1982.05–1982.55	1954.74 ± 0.93 1981.01 ± 0.60 1982.30 ± 0.25
6	−19.5	111.51 ± 0.21	1958.30–1958.60 1995.12–1996.98	4.98	1953.32–1953.62 1990.14–1992.00	1953.47 ± 0.15 1991.07 ± 0.93
7	−16.1	136.10 ± 0.25	1962.36–1962.70 1975.90–1976.88	5.33	1957.03–1957.37 1970.57–1971.55	1957.20 ± 0.17 1971.06 ± 0.49
8	−21.9	85.42 ± 0.20	672–777 792–802 810–820	4.99	667.01–772.01 787.01–797.01 805.01–815.01	ND
9	−16.0	106.58 ± 0.22	1957.32–1957.68 2003.96–2006.00 2006.30–2006.92	4.98	1952.34–1952.70 1998.98–2001.02 2001.32–2001.94	1952.52 ± 0.18 2000.00 ± 1.02 2001.63 ± 0.31
10	−15.2	128.42 ± 0.24	1961.96–1962.42 1979.72–1980.12 1980.46–1980.78	4.98	1956.98–1957.44 1974.74–1975.14 1975.48–1975.80	1957.21 ± 0.23 1974.94 ± 0.20 1975.64 ± 0.16
11	−16.5	111.14 ± 0.26	1958.16–1958.58 1995.90–1997.74 1998.60–1998.72	4.99	1953.17–1953.59 1990.91–1992.75 1993.61–1993.73	1953.38 ± 0.21 1991.83 ± 0.92 1993.67 ± 0.06
12	−18.4	97.68 ± 0.24	1660.82–1687.30 1730.90–1789.96 1790.98–1807.48 1925.76–1954.22	5.33	1655.49–1681.97 1725.57–1784.63 1785.65–1802.15 1920.43–1948.89	1934.66 ± 14.23
13	−16.9	135.97 ± 0.29	1962.40–1962.62 1975.92–1976.86	5.33	1957.07–1957.29 1970.59–1971.53	1957.18 ± 0.11 1971.06 ± 0.47
14	−18.7	96.73 ± 0.25	1524.98–1559.78 1565.20–1571.10 1631.62–1665.10 1785.22–1795.14 1949.20–1953.24	5.33	1519.65–1554.45 1559.87–1565.77 1626.29–1659.77 1779.89–1789.81 1943.87–1947.91	1945.89 ± 2.02
15	−17.5	126.22 ± 0.28	1961.88–1962.28 1980.72–1982.08	5.33	1956.55–1956.95 1975.39–1976.75	1956.75 ± 0.20 1976.07 ± 0.68

Measured values of ^14^C and δ^13^C analyses are shown with the calibrated formation range and tooth formation time (TFT). In this study, the calibrated formation range was defined as the estimated tooth YOB. The estimated YOB range was calculated by subtracting TFT from the estimated tooth YOB. The estimated YOB was calculated using the same practical approach as described by Kunita et al.^26^, where the representative value was defined as (upper limit + lower limit)/2 and the width as (upper limit − lower limit)/2. For no. 8, the estimated YOB range was inconsistent with the condition of the remains and thus was excluded; accordingly, no estimated YOB is presented. For nos. 12 and 14, estimated YOB values outside the 1900 s were considered inappropriate and were excluded.

The estimated tooth YOBs for case no. 8 were 672–777, 792–802, and 810–820; however, these were excluded because they were inconsistent with the condition of the remains and the last confirmed date of survival. Therefore, an estimated YOB was not calculated. Because these calibrated ranges were substantially older than the actual YOB of this individual, the result for case no. 8 was considered anomalous.

For case nos. 12 and 14, the calibrated results included pre-modern calendar ranges that were inconsistent with the forensic information. These ranges were excluded and the remaining ranges were used to estimate the YOBs. The estimated YOBs were 1934.66 ± 14.23 for case no. 12 and 1945.89 ± 2.02 for case no. 14.

### AAR analysis

The results of the calibration curve for specimens aged 25–72 years with known ages are shown in Supplementary Fig. S2 (*r* = 0.95). The estimated ages at death calculated using these data are shown in Table 3. For case no. 2, no valid peaks were detected in the AAR analysis, and the estimated age at death could not be calculated. The absolute error between the estimated age at death and AAD was − 5.82 ± 6.87 years (mean absolute error (MAE) = 7.93 years), and a positive correlation was observed between the two ages (*r* = 0.86).

**Table 3:** Estimated age at death derived from amino acid racemization (AAR) analysis, estimated year of birth (YOB) derived from ^14^C analysis, and the resulting estimated year of death.

Case no.	Estimated age at death	Estimated YOB	Estimated year of death
1	57.71	1955.33 1979.11	2013.04 2036.82
2	ND	1963.57	—
3	53.47	1950.42	2003.89
4	48.91	1965.20 1966.48	2014.11 2015.39
5	49.41	1954.74 1981.01 1982.30	2004.15 2030.42 2031.71
6	54.07	1953.47 1991.07	2007.54 2045.14
7	40.66	1957.20 1971.06	1997.86 2011.72
8	56.10	ND	—
9	37.97	1952.52 2000.00 2001.63	1990.49 2037.97 2039.60
10	43.62	1957.21 1974.94 1975.64	2000.83 2018.56 2019.26
11	33.26	1953.38 1991.83 1993.67	1986.64 2025.09 2026.93
12	50.58	1934.66	1985.24
13	54.46	1957.18 1971.06	2011.64 2025.52
14	51.91	1945.89	1997.80
15	38.44	1956.75 1976.07	1995.19 2014.48

The estimated year of death was calculated by adding the estimated age at death to the estimated YOB. For no. 2, no valid result was obtained from AAR analysis, and for no. 8, no valid result was obtained from the ^14^C analysis; therefore, the estimated year of death could not be determined.

### Narrowing the estimated YOB based on the results of ^14^C analysis and AAR analysis

The estimated year of death was calculated by adding the estimated age at death, derived from AAR analysis, to the estimated YOB obtained from ^14^C analysis (Table 3). For case no. 1, the estimated YOBs of 1955.33 and 1979.11 were combined with the estimated age at death of 57.71 years derived from AAR analysis, resulting in estimated years of death of 2013.04 and 2036.82, respectively. Considering consistency with the last confirmed date of life and the date of autopsy based on investigative information, the final estimated YOB for this case was determined to be 1955.33. The estimated year of death was calculated using the same method for the other samples, and the final estimated YOB was determined (Table S1).

**Fig. 3:**
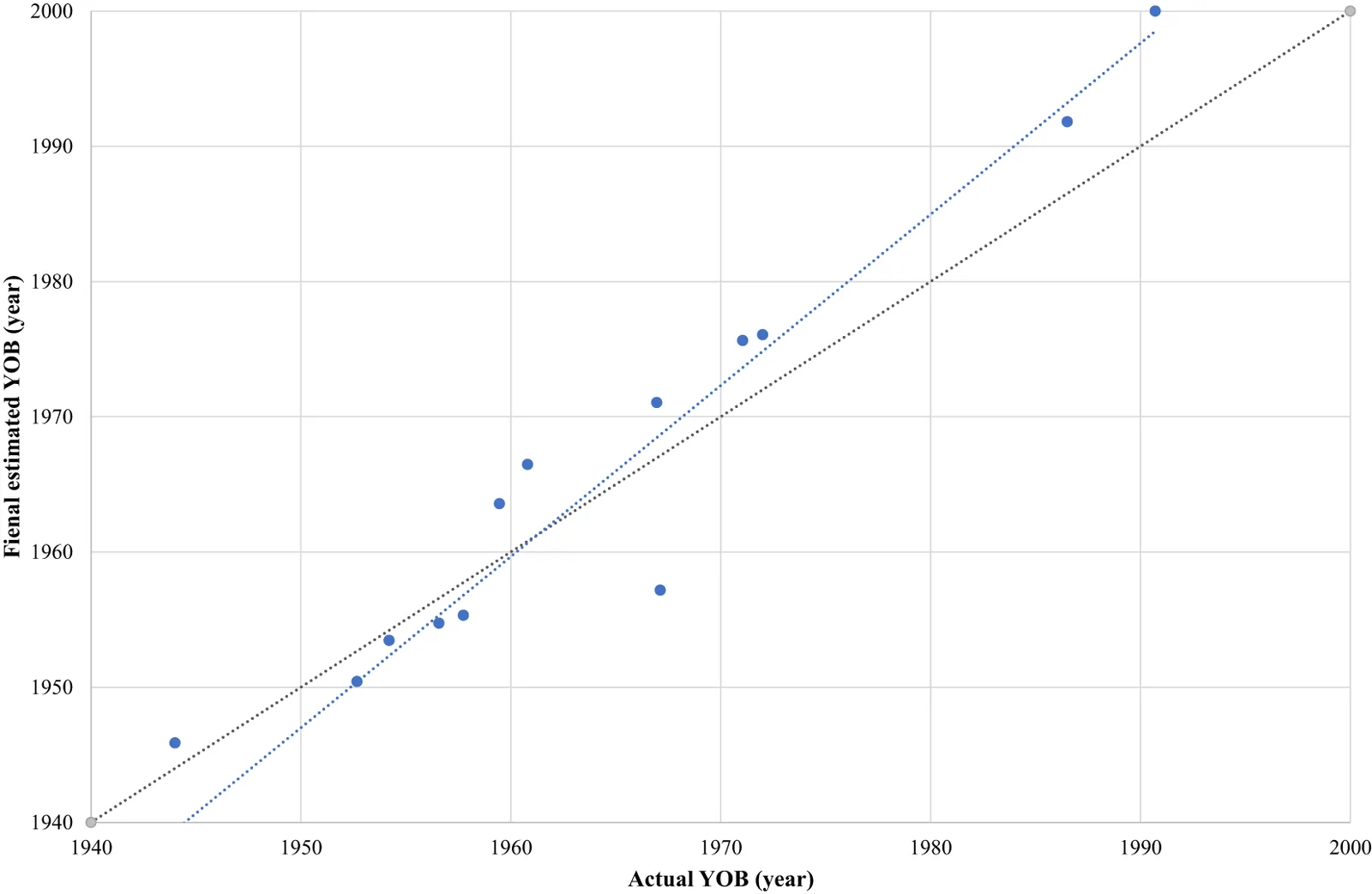
Relationship between final estimated year of birth (YOB) and actual YOB. A strong positive correlation was observed (*r* = 0.95). The gray dashed line represents the line of identity (*y* = *x*).

The mean error between the final estimated YOB and the actual YOB was 0.57 ± 6.36 years (MAE = 5.01 years), and a positive correlation was observed between the two YOBs (*r* = 0.95) (Fig. 3). In the relationship with YOB, smaller errors tended to be observed for YOBs around 1955. The error was 2.41 years for case no. 1 (born in 1957), 1.83 years for case no. 5 (born in 1956), and 0.73 years for case no. 6 (born in 1954). In contrast, the error was 9.30 years for case no. 9 (born in 1990), 13.93 years for case no. 12 (born in 1948), and 9.94 years for case no. 13 (born in 1967), which were much larger errors. These results indicated that samples from individuals born before the rise of the bomb curve and in more recent years tended to exhibit larger estimation errors.

### δ^13^C analysis

The δ^13^C analysis results are shown in Table 2. All samples fell within the range of − 21.9‰ to − 15.2‰, with a mean of − 17.8‰. The mean δ^13^C values by birth decade were − 18.6‰ for those born in the 1940 s (*n* = 2), − 18.9‰ for those born in the 1950 s (*n* = 6), − 16.8‰ for those born in the 1960 s (*n* = 3), and − 16.3‰ for those born in the 1970 s (*n* = 2). There was only one subject each born in the 1980 s and 1990 s, with values of − 16.5‰ and − 16.0‰, respectively. The mean δ^13^C values tended to increase in more recent birth decades, from − 18.6‰ in individuals born in the 1940 s to − 16.0‰ in the individual born in the 1990s.

### DNA analysis

Sex determination was possible for all cases using the three sex chromosome markers included in the GlobalFiler (Y-indel, Amelogenin, and DYS391) and Y-Filer Plus PCR Amplification Kits, and the results were consistent with the postmortem information. Paternal genetic information was obtained from Y-STR analysis, and maternal genetic information from mtDNA analysis.

Regarding autosomal STR typing, profiles with nine or more loci were detected in 12 of the 15 cases, yielding profiles sufficient for individual identification. Detailed results are shown in Table 4. As an example, part of the STR and Y-STR typing results for case no. 5 are shown in Supplementary Fig. S3. The DNA concentrations of the three cases with fewer than nine loci (case nos. 6, 7, and 10) were extremely low, ranging from 0.55 to 0.80 pg/µL. In contrast, the DI values of all DNA extracted from each sample ranged from 0.47 to 1.90, and the IPC Ct values ranged from 27.34 to 33.68, indicating that the effects of substantial DNA degradation or PCR inhibitory substances were minimal.

**Table 4:** Results of DNA quantification by real-time PCR, and results of STR, Y-STR, and mtDNA analyses.

Case no.	Quantity (ng/µL)	DI	IPC Ct	Detection rate of autosomal STRs (%) (number of detected loci)	Detection rate of Y-STR (%)	mtDNA HV1 substitutions (*n*)	mtDNA HV2 substitutions (*n*)
1	0.11822	0.72770	27.69464	95.2 (20)	100.0	2	Not detected
2	0.01021	0.91220	27.46282	100.0 (21)	96.0	6	2
3	0.00980	0.57872	27.83947	100.0 (21)	100.0	1	2
4	0.01077	1.25151	27.57713	85.7 (18)	Not detected	Not tested	Not tested
5	0.04129	0.68748	27.66720	100.0 (21)	100.0	Not detected	7
6	0.00066	0.76091	27.59807	23.8 (5)	—	2	Not detected
7	0.00055	1.60158	27.69042	23.8 (5)	Not detected	2	3
8	0.02150	0.47160	27.74483	100.0 (21)	—	3	3
9	0.00175	1.90284	27.34490	57.1 (12)	—	1	2
10	0.00080	0.80839	27.53761	28.6 (6)	—	2	Not detected
11	0.01106	0.93004	27.63708	76.2 (16)	—	2	1
12	0.00177	0.53896	27.65015	95.2 (20)	80.0	2	1
13	0.00775	1.43637	27.57688	95.2 (20)	96.0	2	1
14	0.13196	1.03595	28.34052	95.2 (20)	80.0	2	Not detected
15	0.00650	1.68480	33.68694	100.0 (21)	96.0	3	3

This table shows, for each sample, the DNA quantity, degradation index (DI), internal PCR control (IPC) Ct value, autosomal STR detection rate (%), number of detected loci, Y-STR detection rate (%), and the number of substitutions in the mtDNA HV1 and HV2 regions. Y-STR analysis was conducted on 10 samples (case nos. 1–5, 7, and 12–15), which were determined to be male by STR profiling.

Of the 10 samples (case nos. 1–5, 7, and 12–15) subjected to Y-STR analysis, valid loci were confirmed in eight samples, excluding case nos. 4 and 7, with detection rates ranging from 80% to 100% for each sample. Regarding mtDNA typing, because all available material for case no. 4 had been used up in the STR typing and Y-STR analysis, the analysis was conducted on the remaining 14 samples. No valid nucleotide sequences were obtained for case no. 5 in the HV1 region, or for case nos. 1, 6, 10, and 14 in the HV2 region. Table 4 shows the number of substitutions in the mtDNA for the samples from which nucleotide sequences were obtained. The detection range and substitution sites are shown in the Supplementary Information (Tables S2 and S3).

### Toxicological analysis

Drugs were detected in all cases (case nos. 1–15). The details, including the samples used for toxicological analysis at the time of autopsy, the results, and information on drug use prior to death, are summarized in Table 5. Screening tests using samples such as blood and femoral muscle were performed in 12 cases at the time of autopsy. The toxicological analysis conducted at the time of autopsy was performed by a separate institution, and the target substances differed in part. Information about drug use before death was obtained in seven cases (nos. 4, 5, 7, 9, 11, and 13) by reviewing medication records and searching living quarters. In the remaining eight cases, no relevant information was obtained, or the individuals were not taking any medication.

**Table 5:** Drugs detected in teeth, drugs detected by toxicological screening at autopsy, and antemortem drug use information identified through investigation.

Case no.	Drugs detected in this study	Samples used for toxicological analysis at autopsy and the detected drugs	Antemortem drug information
1	Caffeine	Caffeine (descending aortic blood, urine)	Not available
2	Caffeine, nicotine, cotinine	Not tested	Not available
3	Caffeine, nicotine, cotinine	Not detected (femoral muscle, liver)	Not available
4	Caffeine, nicotine, cotinine	Caffeine, losartan, theophylline (urine)	Amlodipine, losartan
5	Caffeine, maprotiline, sulpride	Maprotiline, sulpride, diazepam, haloperidol (femur, skull)	Sulpride, haloperidol, cloxazolam, trihexyphenidyl, bromazepam, triazolam, nitrazepam
6	Caffeine	Not tested	Not available
7	Caffeine, amlodipine, bromazepam, chlorpheniramine, dihydrocodeine, irbesartan, methylephedrine, sertraline, loxoprofen, ephedrine	Caffeine, amlodipine, bromazepam, brotizolam, chlorphrniramine, dihydrocodeine, irbesartan, methylephedrine, sertraline (femoral muscle)	Sertraline, dihydrocodeine, brotizolam,
8	Caffeine	Caffeine, dextromethorphan, dextrophan, diprophylline, beta-phenethylamine (femoral muscle)	Not available
9	Caffeine, quetiapine, zotepine	Caffeine, quetiapine, zotepine, diphenhydramine, salicylamide (femoral muscle)	Zotepine, sertraline, nitrazepam, sulpiride, salicylamide, acetaminophen, promethazine
10	Caffeine	Caffeine, beta-phenethylamine (femoral muscle)	Not available
11	Caffeine, chlorpheniramine, etizolam, quetiapine, trazodone, flunitrazepam	Chlorpheniramine, etizolam, quetiapine, trazodone, phenethylamine (right pleural fluid)	Etizolam, quetiapine, trazodone, risperidone, flunitrazepam
12	Caffeine, nicotine, cotinine	Not tested	Not available
13	Caffeine, nicotine, cotinine, sertraline	Sertraline, zopiclone, triazolam (femoral muscle, gastric contents)	Sertraline, zopiclone
14	Caffeine, cotinine	Acetaminophen (femur), not detected (skull)	Not available
15	Caffeine, cotinine	Not detected (aortic blood)	Not available

Drugs detected in teeth, the samples used for toxicological screening at autopsy and the detected drugs, and information on antemortem drug use are presented. In the antemortem drug information, drugs not included in the 251 analytes covered by the present analytical system are not shown in this table.

In none of the cases did the drugs detected in the teeth cover all of the substances identified in the postmortem toxicological screening at autopsy. However, in nine cases (nos. 1, 4, 5, 7–11, and 13), the drugs extracted from the teeth matched some of the results from the screening tests. There were five cases where the information about medications taken during the patient’s lifetime matched the drugs detected in the teeth: sulpiride in case no. 5; sertraline and dihydrocodeine in case no. 7; zotepine in case no. 9; etizolam, quetiapine, trazodone, and flunitrazepam in case no. 11; and sertraline in case no. 13.

## Discussion

There are numerous cases in which, despite investigative efforts, the identity of the deceased remains unidentified. In particular, with highly decomposed bodies or skeletal remains, information from clothing and personal belongings is limited, and the number of leads is greatly reduced owing to the degradation of biological samples caused by postmortem changes. In such situations, teeth, which are structurally the most robust and best-preserved biological tissue, are valuable samples. In this study, we investigated whether the combination of ^14^C and δ^13^C analyses, AAR analysis, DNA typing, and toxicological analysis of a mandibular first premolar could be used to narrow down and identify an individual from an actual forensic case involving advanced postmortem changes. The results demonstrated that all analyses could be performed using a single tooth and that integrating the results of each analysis provided more comprehensive information for personal identification.

^14^C analysis yielded multiple estimated YOBs depending on the individual’s birth year (Table 2). However, combining these results with the estimated age at death derived from AAR analysis enabled year closer to their actual YOB to be selected. Consequently, the estimation error was reduced to 0.57 ± 6.36 years (MAE = 5.01 years). In case no. 15, for which identification has not been achieved, ^14^C analysis gave an estimated YOB of 1956.75 or 1976.07. AAR analysis calculated the age at death as 38.44 years. Based on these results, the estimated year of death was determined to be 1995.19 or 2014.51. Because this was a highly decomposed case, and considering the consistency with the last confirmed date of life (March 2013) and the date of autopsy (April 2013), the final estimated YOB was determined to be 1976.07. This suggests the possibility that the individual was a male born in 1976, who was 38 years old. Currently, the investigation suggests that the individual may have been a man born in 1972, who was 41 years old at the time. Thus, combining information on YOB from ^14^C analysis and the age at death from AAR analysis allows the individual’s year of death to be estimated with high precision, providing a useful clue for identification.

Several issues were identified during the analysis. First, the estimated YOB and ages at death showed slightly larger errors and greater variability than previous studies^10,14^ in which each analysis was conducted separately. One contributing factor is that the ^14^C analysis in this study included subjects born across a wide range of birth years, from the 1940 s to the 1990s. This period encompasses the time before and after the bomb peak from nuclear testing. Because changes in atmospheric ^14^C concentrations were gradual before the peak and minimal more recently, the margin of error in estimating YOB may have been large. In addition to these limitations, the result for case no. 8 was anomalous because the calibrated ranges were substantially older than expected from the known YOB and forensic information. This sample showed the highest carbon content measured by elemental analysis (2.44%; range for all samples, 1.03%–2.44%). Although the carbon content alone is not sufficient to identify the cause of the anomalous result, Wood et al^42^. reported that exogenous carbonate can affect ^14^C dating of tooth enamel. Therefore, exogenous carbon may have contributed to the anomalous ^14^C result in case no. 8. Regarding AAR analysis, the evaluation of estimation accuracy is limited in this study because the actual age at death is unknown. Higher accuracy is typically achieved by analyzing sections from the central part of the dentin^14,15^; however, in this study, we used dentin sections from the outer region rather than the central part because the samples had to be divided for other analyses. These factors may have affected the accuracy of the estimates. When conducting multiple analyses on the same tooth, it will be necessary in future to examine sample collection sites in the tooth and analytical conditions in greater detail.

In δ^13^C analysis, the δ^13^C value of enamel measured during the ^1^⁴C analysis process is thought to reflect the dietary environment during the crown formation period. A diet primarily consisting of C3 plants gives values that are more negative than a diet containing large amounts of C4 plants, such as corn^43^. The δ^13^C values obtained in this study all showed large negative values ranging from − 21.88‰ to − 15.22‰ (Table 2), consistent with a dietary environment dominated by C3 plants, such as rice, which is common in the traditional Japanese diet. This suggests that the subjects in this study were likely exposed to a C3 plant-dominated dietary environment during the crown formation period. δ^13^C values tended to increase gradually in more recent birth decades, possibly reflecting generational changes in dietary carbon sources. However, because the number of samples in each decade was limited, this trend should be interpreted with caution. In addition, because detailed information about place of birth or region of residence during the enamel formation period was unavailable, dietary environments or regions of upbringing could not be accurately inferred based solely on δ^13^C values. To enable more precise regional estimation, analyses incorporating other isotopic indicators, such as oxygen, nitrogen, and strontium, should be conducted^43,44^.

In DNA analysis, high-quality DNA was obtained from the cementum contained in the outermost section, yielding useful STR typing results. Cementum is known to exhibit excellent DNA preservation, and previous studies have reported that sufficient amounts of DNA can be obtained even compared with DNA-rich petrous bones^22^. Consistent with these findings, the present study demonstrated that sufficient DNA can be extracted even from a single longitudinal section of a tooth containing cementum. Thus, DNA typing, including sex determination, can be performed on teeth, to identify individuals quickly. In mtDNA typing, there were cases where mtDNA amplification could not be achieved even in samples that yielded good results in STR analysis. This may be consistent with reports indicating that while cementum preserves nuclear DNA well, the preservation of mtDNA is relatively poor^45^.

In toxicological analysis, substances consistent with conventional toxicological screening results obtained from blood, muscle, and femur samples, as well as with antemortem medication records, were detected even from a small amount (approximately 100 mg) of tooth fragment samples. In particular, some cases suggested that drugs believed to have been taken long-term, such as antipsychotics and antihypertensives, could also be detected in teeth. These results indicate that teeth may reflect a history of habitual or long-term drug use. In cases suggesting acute drug intake immediately before death, although drugs were detected in gastric contents and muscle tissue, they were not detected in dentin or dental pulp. These findings suggest that drug transfer to teeth may be slow, implying that dental samples may be more useful as indicators of long-term drug exposure rather than short-term drug use. In cases where the time since death is prolonged, it is often difficult to collect conventional analytical samples, such as soft tissues or body fluids; however, this study demonstrated that teeth may serve as a useful alternative sample for postmortem toxicological analysis in such situations.

In this study, tooth samples provided diverse information, including estimated YOB, stable isotope-based dietary information, estimation of age at death, DNA typing, and history of drug use. Therefore, even in bodies with prolonged postmortem intervals, a single tooth can be a useful analytical target for personal identification. We intend to increase the number of cases, optimize sample collection sites and analytical conditions, and clarify the relationship with other isotope analyses and pharmacokinetics, thereby clarifying the forensic utility of this method.

## Conclusion

We performed radiocarbon (^14^C) and stable isotope (δ^13^C) analyses, AAR analysis, DNA typing, and toxicological analysis on mandibular first premolars from bodies with prolonged postmortem intervals. A single tooth provided various information, including estimated YOB, dietary habits during enamel formation and the associated regional origin, estimated age at death, sex, DNA genotype, paternal and maternal genetic lineages, and information on drugs and toxic substances. The findings of this study demonstrate that, not only in large-scale disasters but also in all cases when handling unidentified remains, in addition to conventional examinations, collecting and preserving even a single tooth and applying a variety of biochemical analysis to that tooth is an important means of increasing the likelihood of identification.

## Supplementary Information

Below is the link to the electronic supplementary material.

Supplementary Material 1

## Data Availability

The data are not publicly available due to ethical and privacy restrictions but are available from the corresponding author upon reasonable request.
