# Supplementary material for: Multiple forensic insights for human identification using single teeth with applicability to advanced postmortem remains
Source: Sci Rep. 2026 Aug 19;16:25134. doi: 10.1038/s41598-026-62088-z (PMC13490551; doi:10.1038/s41598-026-62088-z)
Supplement: Supplementary file 1 [file 41598_2026_62088_MOESM1_ESM.pdf]

Supplementary Information

Multiple forensic insights for human identification using single teeth with applicability to advanced postmortem remains

Sayaka Yamada[1*], Hisako Saitoh[1*], Kanju Saka[2], Saki Minegishi[1], Toru Moriya[3], Mirei Takeyama[3], Hajime Utsuno[1], Shuuji Namiki[1], Muhammad Garry Syahrizal Hanafi[1], Nanami Aoki[1], Yohsuke Makino[2], Hirotaro Iwase[4], Fuyuki Tokanai[3], Koichi Sakurada[1]

[1]Department of Forensic Dentistry, Graduate School of Medical and Dental Sciences, Institute of Science Tokyo, 1-5-45 Yushima, Bunkyo-ku, Tokyo 113-8510, Japan

[2]Department of Forensic Medicine, Graduate School of Medicine, The University of Tokyo, 7-3-1 Hongo, Bunkyo-ku, Tokyo 113-0033, Japan

[3]Center for Accelerator Mass Spectrometry, Yamagata University, 19-5 Kanakame, Kaminoyama, Yamagata 999-3101, Japan

[4]Department of Legal Medicine, Graduate School of Medicine, Chiba University, 1-8-1 Inohana, Chuo-ku, Chiba 260-8670, Japan

*Corresponding author. Email: yamada.sayaka@tmd.ac.jp, saitoh.hisako@tmd.ac.jp

Contents

Supplementary Figs. S1–S3

Supplementary Tables S1–S3

Supplementary Fig. S1 Serial longitudinal sections approximately 1 mm thick were prepared according to the size of each tooth. Enamel was removed from each section.

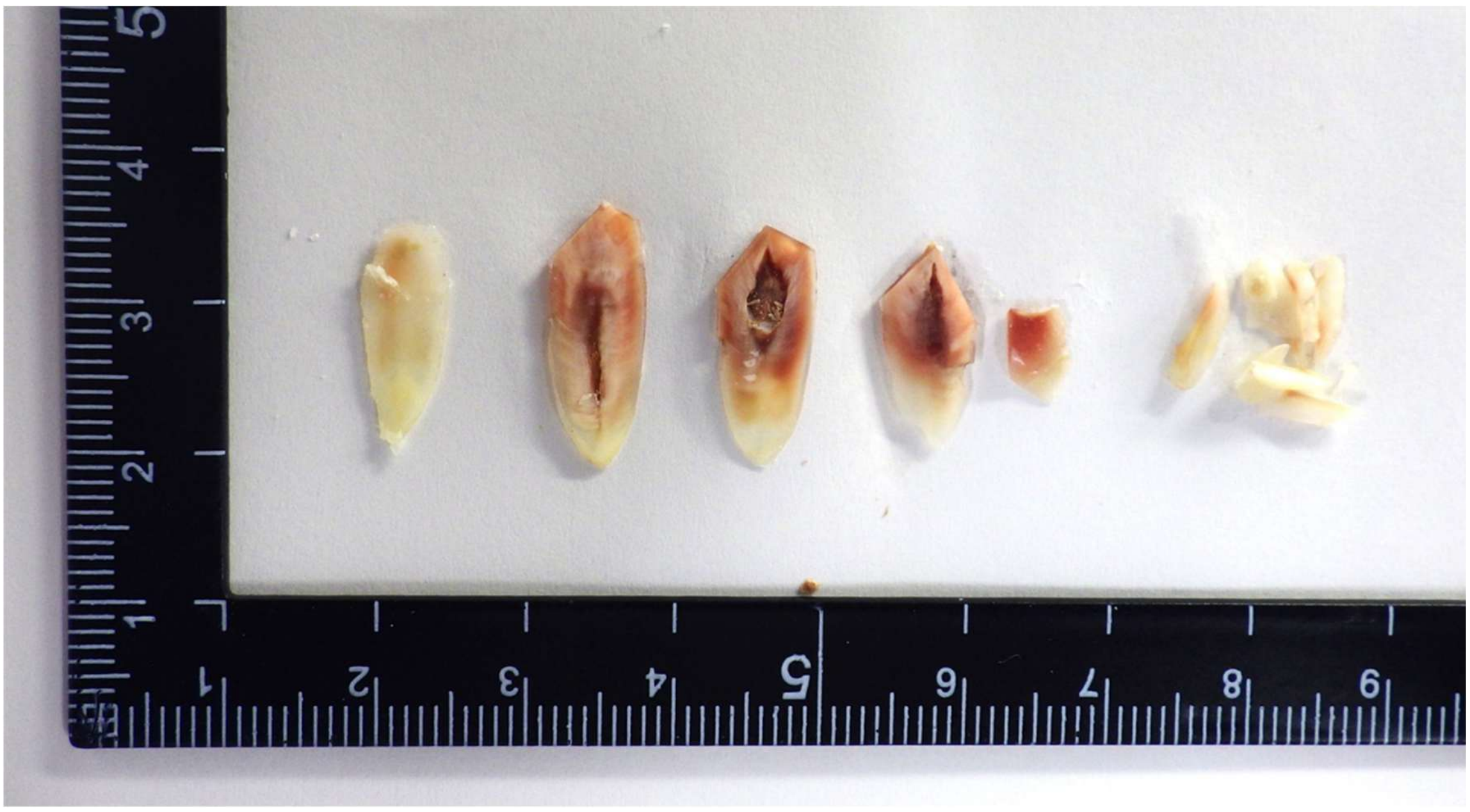

Supplementary Fig S2 Calibration curve used for amino acid racemization analysis. We measured the rate of racemization in the mandibular first premolars of individuals aged 25 to 72. Age and racemization rate showed a strong positive correlation ($r = 0.95$).

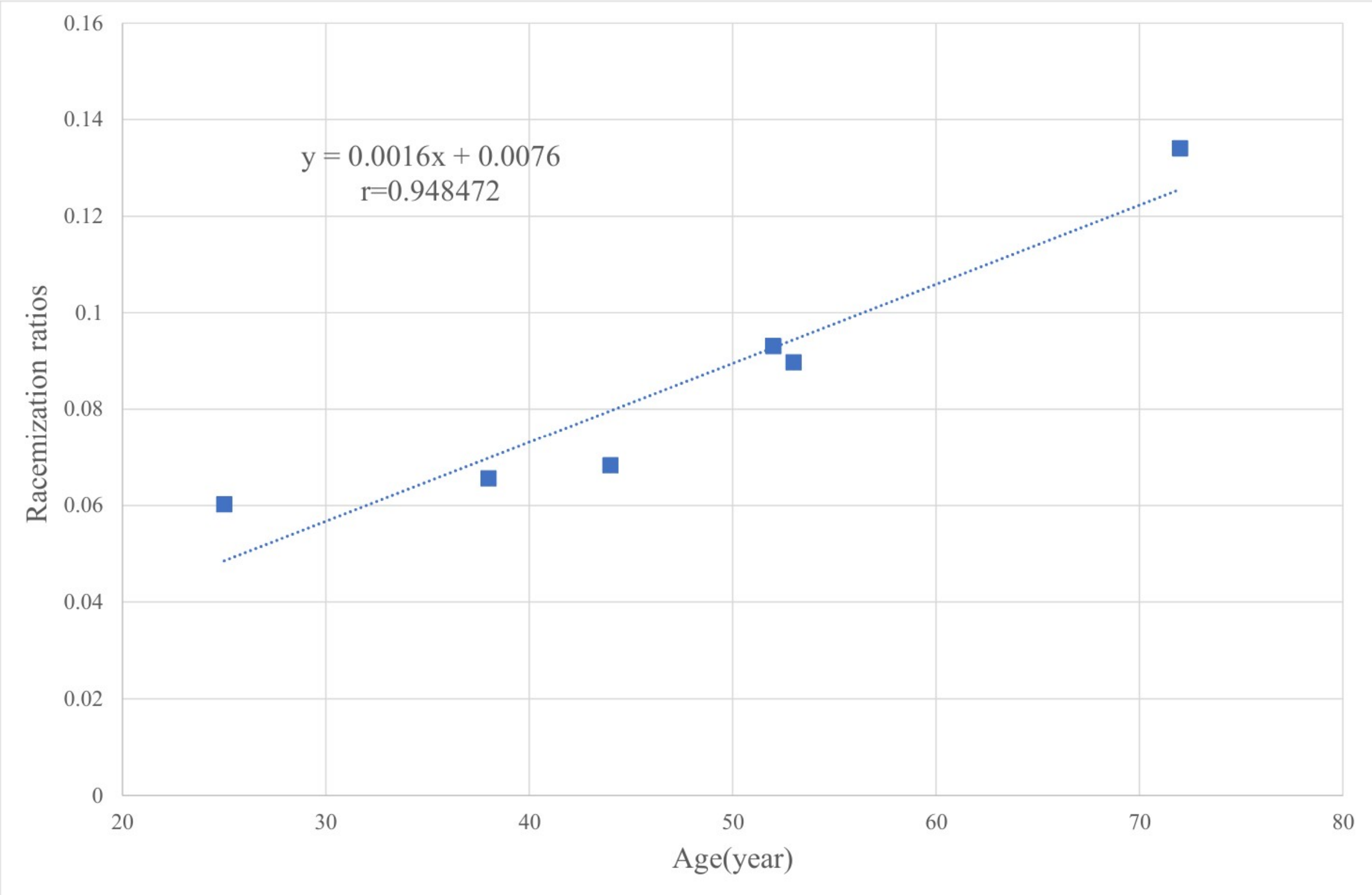

Supplementary Fig. S3 Example of STR typing and Y-STR results. Partial analysis results for sample no. 5 are shown below. a: STR typing. b: Y-STR typing.

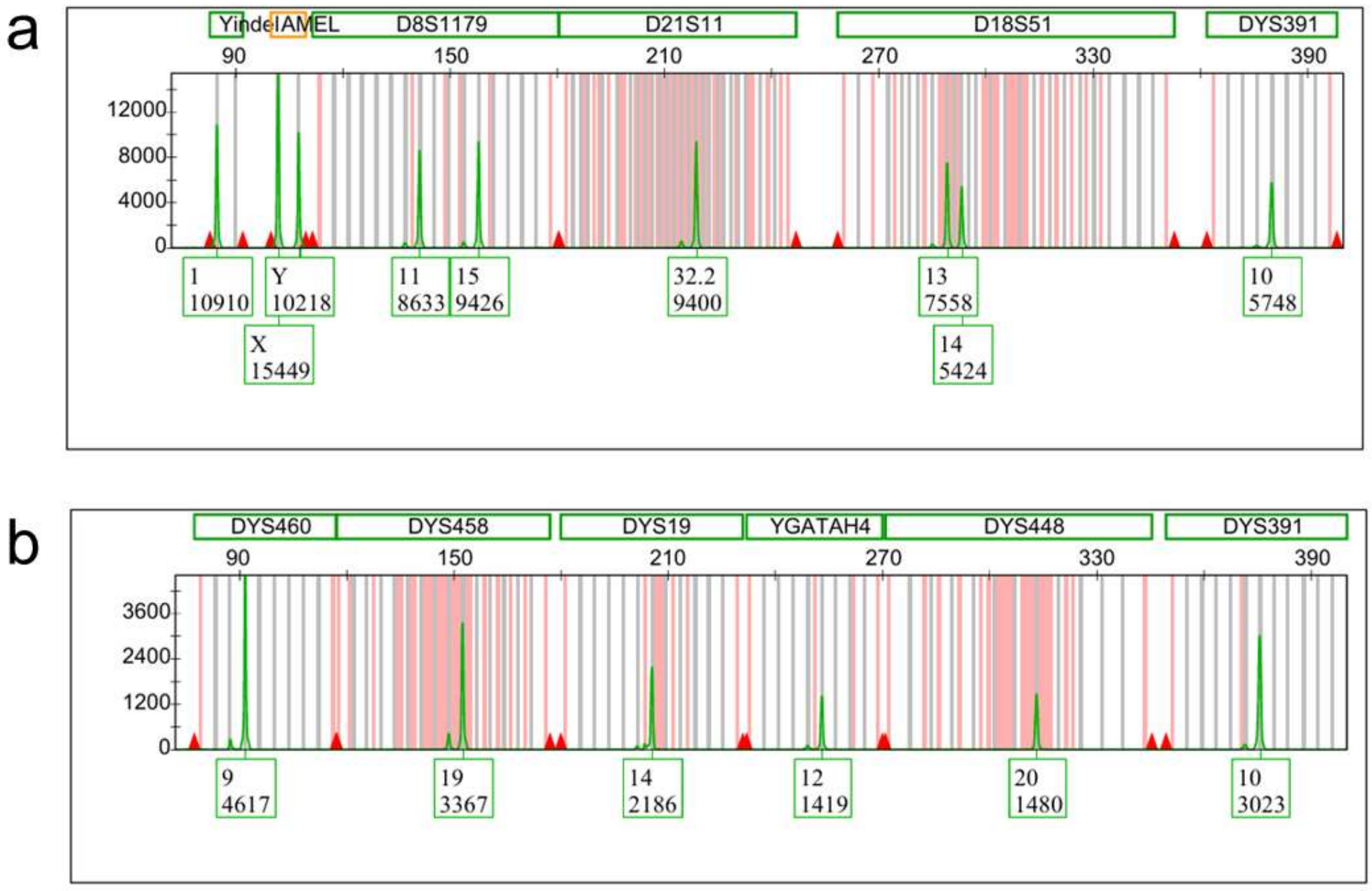

Supplementary Table S1 Comparison of Final estimated year of birth (YOB) and Actual YOB with absolute error.

Case no.	Actual YOB	Final estimated YOB	Absolute error
1	1957.74	1955.33	2.41
2	1959.45	1963.57	4.12
3	1952.67	1950.42	2.25
4	1960.80	1966.48	5.68
5	1956.57	1954.74	1.83
6	1954.20	1953.47	0.73
7	1966.95	1971.06	4.11
8	1952.32	ND	ND
9	1990.70	2000.00	9.30
10	1971.04	1975.64	4.60
11	1986.51	1991.83	5.32
12	1948.59	1934.66	13.93
13	1967.12	1957.18	9.94
14	1944.00	1945.89	1.89
15	1972	1976.07	4.04

The estimated YOB derived from ^{14}C analysis and AAR analysis is presented as the final estimated YOB, and the difference between the actual YOB and the final estimated YOB is presented as the margin of error.

For no. 15, because the date of birth of the leading candidate for the identity was unknown, actual YOB was calculated by assuming 1972 based on their age at the time (41 years old).

Supplementary Table S2 Results of mtDNA HV1 region analysis.

Case no.	Range	Haplotype
1	16,086–16,195	16111T 16189C
2	16,086–16,228	16189C 16194G 16195C 16223T 16223T 16227G
3	16,086–16,254	16223T
4	Not performed	Not performed
5	Not detected	Not detected
6	16,086–16,255	16209C 16223T
7	16,086–16,255	16129A 16223T
8	16,086–16,254	16129A 16189Y 16223T
9	16,086–16,255	16223T
10	16,086–16,254	16187T 16223T
11	16,086–16,255	16209C 16223T
12	16,086–16,201	16111T 16189C
13	16,086–16,193	16111T 16189C
14	16,086–16,254	16209C 16223T
15	16,086–16,193	16182C 16183C 16189C

Base pair ranges and haplotypes of the mtDNA HV1 region are shown for each sample.

Supplementary Table S3 Results of mtDNA HV2 region analysis.

Case no.	Range	Haplotype
1	Not detected	Not detected
2	97–340	263G 315.1C
3	109–367	263G 315.1C
4	Not performed	Not performed
5	96–315	103A 152C 199C 203A 204C 263G 309.1C
6	Not detected	Not detected
7	96–360	152C 263G 315.1C
8	97–318	263G 309.1C 315.1C
9	97–320	263G 315.1C
10	Not detected	Not detected
11	295–380	315.1C
12	293–370	315.1C
13	287–340	315.1C
14	Not detected	Not detected
15	111–315	150T 263G 309.2C

Sequence ranges and haplotypes of the mtDNA HV2 region are shown for each sample.
